# Multitarget mechanisms of the herb pair *Achyranthes bidentata* and *Paeonia lactiflora* Pall. in ameliorating hypertensive cardiomyopathy: combining network pharmacology and functional exploration

**DOI:** 10.3389/fphar.2026.1717533

**Published:** 2026-02-11

**Authors:** Yanyan Zhang, Yingwanqi Wang, Jianing Liu, Yu Zhao, Yuhui He, Peimei Yan, Shan Ren, Ying Lyu, Weiwei Jia, Yuran Sun, Song Lin, Yan Lin

**Affiliations:** 1 School of Basic Medicine, Qiqihar Medical University, Qiqihar, China; 2 Department of Medicine, Ningbo University, Ningbo, Zhejiang, China; 3 School of Medical Technology, Qiqihar Medical University, Qiqihar, China; 4 Heilongjiang Provincial Key Laboratory of Food and Medicine Homology and Metabolic Disease Prevention, Qiqihar Medical University, Qiqihar, China

**Keywords:** *Achyranthes bidentata* Blume, hypertensive cardiomyopathy, network pharmacology, *Paeonia lactiflora* Pall., paeoniflorin

## Abstract

The herb pair *Achyranthes bidentata* Blume and *Paeonia lactiflora* Pall. (AB-PL) has demonstrated significant efficacy in the treatment of hypertensive cardiomyopathy; however, its underlying mechanisms remain unclear. In this study, we established an integrated framework combining predictive network pharmacology and experimental exploration to investigate the action mechanisms of AB-PL. The potential antihypertensive targets and pathways of AB-PL were predicted via network pharmacology analysis. These predictions were further explored and assessed in a hypertensive C57 mouse model using real-time quantitative polymerase chain reaction (RT-qPCR), Western blotting, immunohistochemistry, metabolomics, and other assays. The network pharmacology analysis suggested that the active components of AB-PL (e.g., paeoniflorin) may alleviate hypertensive cardiomyopathy by modulating nitrogen metabolism, oxidative stress, inflammation, and the lipid metabolic pathways. Our experimental results show that AB-PL significantly reduces blood pressure and decreases the levels of atrial and brain natriuretic peptides while improving left ventricular ejection fraction in hypertensive mice. AB-PL treatment also ameliorates abnormalities in the nitrogen metabolism biomarkers. Fluorescent probe detection, RT-qPCR, and immunohistochemical analyses indicate that AB-PL significantly alters the expression levels of reactive oxygen species, superoxide dismutase, Keap1, Nrf2, and HO-1. In addition, AB-PL suppresses inflammatory responses by inhibiting the expression of NLRP3, ASC, gasdermin D, IL-1β, and IL-18. Metabolomic analysis further suggested the regulatory effects of AB-PL on the lipid metabolism pathways, including fatty acid biosynthesis. In conclusion, this study provides insights into the potential antihypertensive cardiomyopathic mechanisms of AB-PL through a combined bioinformatic and experimental approach, which support its potential clinical application for the prevention and treatment of hypertensive cardiomyopathy.

## 1 Introduction

Hypertension is a preeminent cardiovascular burden that affects billions of people globally and is intimately associated with life-threatening complications like stroke and heart failure (World Health Organization, 2023). Hypertension-induced myocardial remodeling involves structural and functional changes to the heart muscle, including hypertrophy, fibrosis, and inflammation. These changes can impair both systolic and diastolic functions to increase the risk of cardiovascular events and heart failure (Gaydarski et al., 2025; Huang et al., 2024). Despite recent advancements in antihypertensive therapies, there are persistent treatment challenges where nearly half of the patients exhibit primary resistance to monotherapies, highlighting the urgent need for innovative strategies (Heidari et al., 2022; Tedesco et al., 2006). The polypharmacological properties of traditional Chinese medicine offer promising therapeutic alternatives, but the lack of systematic insights into their multicomponent regulatory mechanisms has hindered clinical translation.

The herb pair *Achyranthes bidentata* Blume and *Paeonia lactiflora* Pall. (AB-PL) has demonstrated significant cardiac protective effects in preclinical models; our previous research showed that paeoniflorin and β-ecdysterone are the major active components of AB-PL that exhibit remarkable therapeutic effects on cardiovascular diseases (Ren et al., 2023; Yan et al., 2025). However, there remains a critical gap in our understanding of AB-Pl in that the existing mechanistic investigations are predominantly confined to single components or isolated signaling pathways, which fail to capture the holistic regulatory effects of AB-PL across interconnected biological networks. Notably, although previous studies have explored the effects of AB-PL on individual physiological processes, none of the studies have systematically evaluated its regulatory impacts on the integrated network of pathways underlying hypertensive pathogenesis. This methodological limitation has precluded a comprehensive understanding of the therapeutic mechanisms of AB-PL.

To address this knowledge void, we established an integrative research framework combining network pharmacology and functional verifications. We employed network pharmacology to predict the potential “component–target–pathway” networks of AB-PL, which were then validated using real-time quantitative polymerase chain reaction (RT-qPCR), Western blotting, immunohistochemistry, and metabolomics. This approach was designed to decode the multilevel regulatory effects of AB-PL by focusing on its potential to modulate interconnected pathways relevant to hypertension.

In this study, we identified the key targets and pathways of AB-PL via network pharmacology, validated its regulatory effects on the physiological processes underlying hypertension, and finally elucidated the holistic therapeutic mechanisms. By addressing the mechanistic ambiguity of AB-PL, this work not only advances the field of herbal medicine research but also provides novel insights for the development of innovative antihypertensive strategies.

## 2 Materials and methods

### 2.1 Collection of metabolite targets of the effective components of AB-PL

The metabolites of the active components in all drug pairs of AB-PL were collected from the TCMSP database (http://lsp.nwu.edu.cn/tcmsp.php) and screened on the basis of oral bioavailability (OB) ≥30% and drug-likeness (DL) ≥0.18; this was determined by taking into consideration the characteristics of oral drugs, which undergo absorption, distribution, metabolism, and excretion (ADME) in the body before exerting their biological effects.

### 2.2 Acquisition of therapeutic targets for hypertension

The potential targets associated with hypertension were screened using the keyword “hypertension” on the TTD (http://db.idrblab.net/ttd/), DisGeNET (http://www.disgenet.org), GeneCards (Dahary et al., 2019; Rappaport et al., 2017) (https://genecards.weizmann.ac.il/v3/), and OMIM (Amberger et al., 2017; https://omim.org/) databases. Furthermore, the compound names were standardized in accordance with PubChem compound identification numbers (https://pubchem.ncbi.nlm.nih.gov/). The obtained targets were submitted to the SwissTargetPrediction website (http://www.SwissTargetPrediction.ch/index.php) for verification of the genetic names.

### 2.3 Acquisition of potential targets for treating hypertension with AB-PL

The overlapping items among the AB, PL, and disease targets were acquired and visualized using the Venn diagram online website (http://bioinformatics.psb.ugent.be/webtools/Venn/). The intersecting items were the core targets of AB-PL against hypertension that were used for further analyses.

### 2.4 Gene Ontology (GO) and Kyoto Encyclopedia of Genes and Genomes (KEGG) pathway enrichment analyses

We performed GO functional and KEGG pathway analyses using the DAVID database. GO analysis provides an in-depth exploration of the associated biological processes (BP). Here, items with *p*-values less than 0.05 were employed for further analyses, and the top-20 GO and KEGG terms with the highest enrichment were selected and visualized.

### 2.5 Protein–protein interaction (PPI) network construction

The overlapping targets between AB-PL and hypertension were imported into the STRING database (https://string-db.org/) to construct the PPI network. Here, the species criterion was set to “*Homo sapiens*” and the confidence score was set to ≥0.700. Next, Cytoscape 3.10.1 software was used to visualize and analyze the PPI network (Otasek et al., 2019).

### 2.6 Experimental verification

#### 2.6.1 Medicines and reagents

Granules of *A. bidentata* Blume (batch no. 1013141; 2.5 g of granules equivalent to the active components in 10 g of stir-fried processed decoction pieces used clinically) and *P. lactiflora* Pall. (batch no. 0129411; 1 g of granules equivalent to 10 g of decoction pieces used clinically) were purchased from Guangzhou Yifang Pharmaceutical Co., Ltd. *Aconitum carmichaelii* Debeaux extract was obtained from Qiqihar Hospital of Traditional Chinese Medicine. The plant names were verified through http://MPNS.kew.org. L-NAME (N-gamma-nitro-L-arginine methyl ester; purity ≥98%; batch no. N5751) was obtained from Sigma-Aldrich (Shanghai Trading Co., Ltd), while fosinopril sodium tablets (batch no. 21001030) were purchased from Shanghai SmithKline Beecham Co., Ltd. (China and United States).

#### 2.6.2 Laboratory animals

Six-week-old male C57BL/6 mice (20 ± 2 g) were procured from the Animal Research Institute of Qiqihar Medical University (experimental animal license no. SYXK, 2021–0013; ethical approval number: QMU-AECC-2022-115). The animals were housed and maintained in the specific-pathogen-free (SPF) animal facility at Qiqihar Medical University. All mice were raised in a well-ventilated SPF-grade room (temperature: 22 °C ± 2 °C; relative humidity: between 45% and 55%; 12/12 h light/dark cycle) and received food and water *ad libitum*. After 1 week of adaptive feeding, the mice were randomly divided into four groups as follows: control group (n = 10), model group (L-NAME, n = 15), pharmacological intervention group (L-NAME + AB-PL, n = 10), and positive control group (L-NAME + fosinopril, n = 10). L-NAME was administered by gavage at a dose of 60 mg/kg (Greish et al., 2020). The AB-PL formulation granules were dissolved in distilled water before administration. The dosing regimen for the treatment groups were calculated based on the original medicinal dosages as follows: AB:PL = 2.73 g/kg: 4.095 g/kg (Yanyan et al., 2024); fosinopril was administered orally at 20 mg/kg/d (Juan et al., 2017). The control group received gavage with the same volume of physiological saline, while the AB-PL group received gavage for 8 weeks concurrently with the L-NAME modeling procedure.

### 2.7 Animal experimentation

#### 2.7.1 Blood pressure measurement

Blood pressure was measured in the experimental animals after 8 weeks of treatment. The measurements were performed by tail-cuff plethysmography using an electrosphygmomanometer (CODA, Kent Scientific, United States) equipped with occlusion cuffs, volume pressure recording cuffs, a restraining cone, and a warming board to keep the animals warm. The animals were habituated prior to the blood pressure measurements, where the systolic, diastolic, and mean arterial pressures were recorded. The average of at least 30 readings obtained in the quiescent state was calculated for each animal. Then, changes in the systolic, diastolic, and mean arterial blood pressure values were analyzed.

#### 2.7.2 Echocardiographic analysis

A Vevo2100 ultrahigh-resolution ultrasound system (VisualSonics, Canada) was used for standard transthoracic echocardiogram analysis after 8 weeks of drug interventions. The left ventricular parameters like ejection fraction and fractional shortening were calculated.

#### 2.7.3 Measurement of the heart to bodyweight and heart weight to tibia length ratios

After 8 weeks of treatment, the mice were anesthetized with tribromoethanol (0.0125 mg/10 g, T48402, Sigma, Germany), and the hearts were removed. The hearts were then rinsed with ice-cold saline and any excess fluid was removed by blotting with filter paper. Simultaneously, the mice tibiae were removed for measurements. Next, the hearts were weighed to calculate the heart to bodyweight and heart weight to tibia length ratios based on the formulas total weight of heart (mg)/total weight of mouse body (g) (HW/BW) and total weight of heart (mg)/tibia length (mm) (HW/TL), respectively. These ratios serve as quantitative indicators of cardiac hypertrophy and may also indirectly reflect the degree of renal fibrosis.

#### 2.7.4 Enzyme-linked immunosorbent assay (ELISA)

The serum levels of endothelial nitric oxide synthase (eNOS), inducible nitric oxide synthase (iNOS), interleukin (IL)-1β, and IL-18 were determined by ELISA using kits from Jianglai (Shanghai, China) according to the manufacturer’s instructions.

#### 2.7.5 Nitric oxide (NO) assay

The serum level of NO was indirectly determined using the total nitric oxide assay kit (S0024, Beyotime, Shanghai, China), which measures the concentrations of nitrates and nitrites by the Griess assay. The optical density at 540 nm was recorded using a multimode detector (SpectraMax iD5, Molecular Devices, Shanghai, China), and the NO concentration was calculated from the standard curve.

#### 2.7.6 Fluorescent staining for detection of reactive oxygen species (ROS)

Myocardial tissues collected from the mice were placed in a centrifuge tube, and homogenization buffer A was added at a tissue-weight-to-buffer-volume ratio of 1:9 (BB-470538, Bestbio, Nanjing, China). The tissues were then fully homogenized with a homogenizer. After incubation at 4 °C, the samples were centrifuged at 100 × *g* for 5 min, and the supernatant was collected and stored for later use. Then, 190 μL of the homogenization supernatant and 10 μL of probe were added to a 96-well plate, mixed well by pipetting, and incubated for 30 min at 37 °C away from light. After incubation, the plate was placed on a microplate reader, and the fluorescence intensity was measured with excitation at 488 nm and emission at 530 nm. Next, 50 μL of the supernatant homogenate diluted approximately 30-fold with phosphate-buffered saline and 100 μL of probe were taken for protein quantification. The ratio of fluorescence intensity to protein concentration was used to represent the ROS level in the tissues.

#### 2.7.7 Superoxide dismutase (SOD) enzyme activity assay

An appropriate amount of mouse cardiac tissue was weighed and homogenized on ice with the SOD sample preparation buffer at a ratio of 10 mg of tissue per 100 μL of buffer. The homogenate was centrifuged at 12,000 × *g* for 5 min at 4 °C, and the supernatant was collected. The protein concentration was determined using the BCA method, and SOD activity was measured according to the instructions of the total SOD activity assay kit (S0101M, WST-8 method, Beyotime, Shanghai, China). In brief, 20 μL of the diluted sample and 160 μL of WST-8/enzyme working solution were added to a 96-well plate, along with 20 μL of the reaction initiation solution. After incubation at 37 °C for 30 min, the absorbance was measured at 450 nm. The percentage of inhibition was calculated based on the difference in absorbances between the blank controls and sample wells, which was then converted into SOD enzyme activity. The results were expressed in units per milligram of protein (U/mg protein).

#### 2.7.8 RT-PCR analysis

The total RNA was isolated from the mice heart tissues using the TRIzol method, and complementary DNA (cDNA) was prepared using the PrimeScript™ RT Reagent kit (TaKaRa, Kusatsu, Japan) according to manufacturer instructions. RT-PCR was then performed in a Light Cycler 480 II Real-Time PCR system (Roche Diagnostics, Basel, Switzerland) using TB Green™ Premix Ex Taq™ (TaKaRa, Kusatsu, Japan). The primer sequences used in this step are shown in Supplementary Table S2. The mRNA level was quantified by the 2^−ΔΔCt^ method and normalized to the mRNA level of GAPDH.

#### 2.7.9 Western blotting

The samples were removed from −80 °C storage and immediately placed on ice. Then, they were minced and homogenized in ice-cold RIPA buffer containing 100 mM of phenylmethylsulfonyl fluoride. The total protein concentration was measured using the BCA assay kit (Boster Biological Technology Co., Ltd., Wuhan, China). The proteins were subjected to SDS-PAGE electrophoresis, transferred to NC membranes, blocked with 5% skimmed milk for 2 h, and incubated overnight at 4 °C with nuclear factor erythroid-2-related factor 2 (Nrf2; Zen bioscience, China), Keap1 (Proteintech, United States), SOD (Proteintech, United States), heme oxygenase-1 (HO-1; Proteintech, United States), NLRP3 (Proteintech, United States), ASC (Proteintech, United States), and caspase-1 (Proteintech, United States). Subsequently, the membranes were incubated with horseradish-peroxidase-conjugated secondary antibodies (ZSGB-Bio, China) for 2 h. The samples were then visualized using an enhanced chemiluminescence advanced kit and a gel imaging system (Tanon Science and Technology Co., Ltd., China).

### 2.8 Metabolomics

Metabolomic analyses of the mouse serum samples were performed using the BioTree method (Shanghai, China). Detailed information (e.g., sample preparation, sample analysis, and data processing) about the metabolomics study is provided in the Supplementary Material.

### 2.9 Statistical analysis

Statistical analysis of the experimental data was performed using GraphPad Prism 8.0.2 software (GraphPad Software, San Diego, CA, United States), ImageJ, and SPSS 19.0 software. Our data were repeated for at least three independent trials. The data are presented as mean ± standard error of the mean (SEM), and an unpaired t-test was used for comparisons between two groups. Statistical significance was indicated for *p-*values <0.05. Image-Pro Plus and ImageJ were used to quantify the positive areas of immunohistochemistry and the Western blotting bands. The heatmap and correlation analyses were performed and plotted using online tools (https://www.bioinformatics.com.cn).

## 3 Results

### 3.1 Pharmacological analysis of the AB-PL network

**FIGURE 1:**
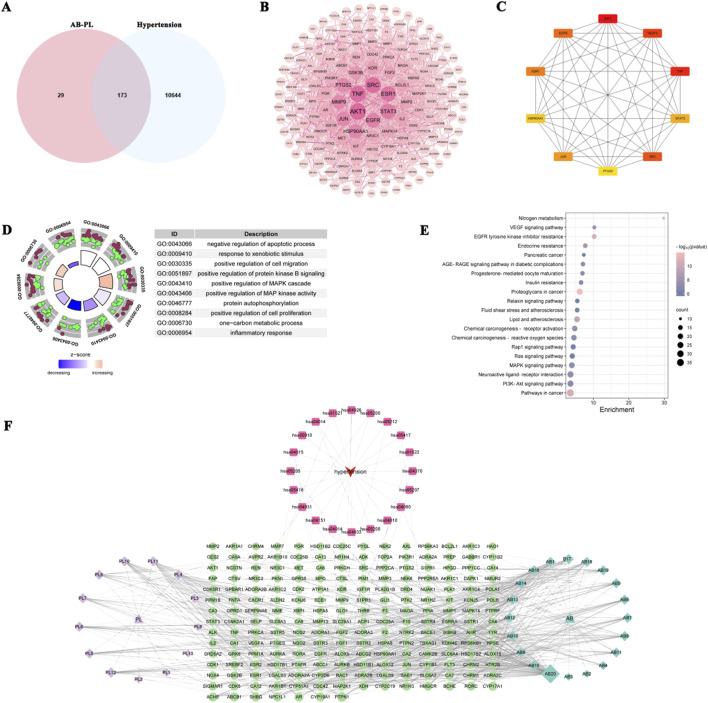
Network pharmacology analysis of the therapeutic targets and pathways of the herb pair *Achyranthes bidentata* Blume and *Paeonia lactiflora* Pall. (AB-PL) in the treatment of hypertension. **(A)** Venn diagram illustrating the overlapping items between hypertension-related genes and potential AB-PL targets. **(B)** Protein–protein interaction (PPI) network of the common targets. **(C)** Important core targets identified from the PPI network. **(D)** Bubble plot showing the top enrichment terms of the Gene Ontology biological processes. **(E)** Bubble plot displaying the top results of the Kyoto Encyclopedia of Genes and Genomes pathway enrichment analysis. **(F)** Network diagram depicting the component–target–pathway interactions: purple and blue squares represent active compounds; purple and blue triangles denote herb names; red arrows indicate hypertension; red squares signify pathways; green circles represent common target genes.

After screening the TCMSP database using the ADME parameters (OB ≥ 30% and DL ≥ 0.18), a total of 33 bioactive ingredients of AB-PL were selected, including 20 ingredients in *A. bidentata* Blume and 13 ingredients in *P. lactiflora* Pall. (Supplementary Figure S1). Among them, kaempferol and beta-sitosterol are two common components. Following the methods described above, 202 component-related targets were obtained after removing duplicate data. A total of 10,817 hypertension-related target genes were collected from the GeneCards, OMIM, and DisGeNET databases. A Venn diagram showed that 173 common targets could be identified by intersecting the targets of AB-PL and hypertension (Figure 1A). The PPI network contained 173 nodes and 1,597 edges (Figure 1B), with the mean degree being 18.5. In the current PPI network, AKT1 antigen (AKT1), tumor-necrosis-like factors (TNF), vascular endothelial growth factor A (VEGFA), and Src tyrosine kinase (SRC) had high degrees of 87, 80, 70, and 68, respectively (Figure 1C). To elucidate the multiple biological functions of AB-PL and the mechanisms by which it improved hypertension, the 173 common targets were imported into DAVID database for GO and KEGG enrichment analyses. The GO plot was used to visualize the corresponding enrichment results, including the top-20 BP items (Figure 1D). For the BPs, the key intersection targets were mainly enriched for inflammatory responses. As shown in Figure 1E, the top-20 hypertension-related pathways enriched for KEGG included inflammatory responses, positive regulation of nitrogen metabolism, EGFR signaling pathway, lipids and atherosclerosis, chemical carcinogenesis, ROS, and PI3K–Akt signaling pathway, among others. The nitrogen metabolism was considered the most important pathway for AB-PL antagonism on hypertension, with the highest target count enrichment and low *p*-value. We also analyzed the network relationship of the important signal pathways of AB-PL for improving hypertension. To construct the component–target–pathway network diagram in Cytoscape 3.10.1 software, we imported the key components, core targets, and signaling pathways of AB-PL (Figure 1F). The results show that there are interconnections between the various pathways and that the active ingredients of AB-PL are more likely to exert synergistic effects by intervening with different signaling pathways to achieve treatment of hypertension. Therefore, in the present study, we selected pathways related to nitrogen metabolism, ROS metabolism, inflammatory responses, and lipid metabolism to investigate the potential mechanisms of AB-PL in the treatment of hypertension.

### 3.2 AB-PL improves blood pressure and cardiac hypertrophy in mice

**FIGURE 2:**
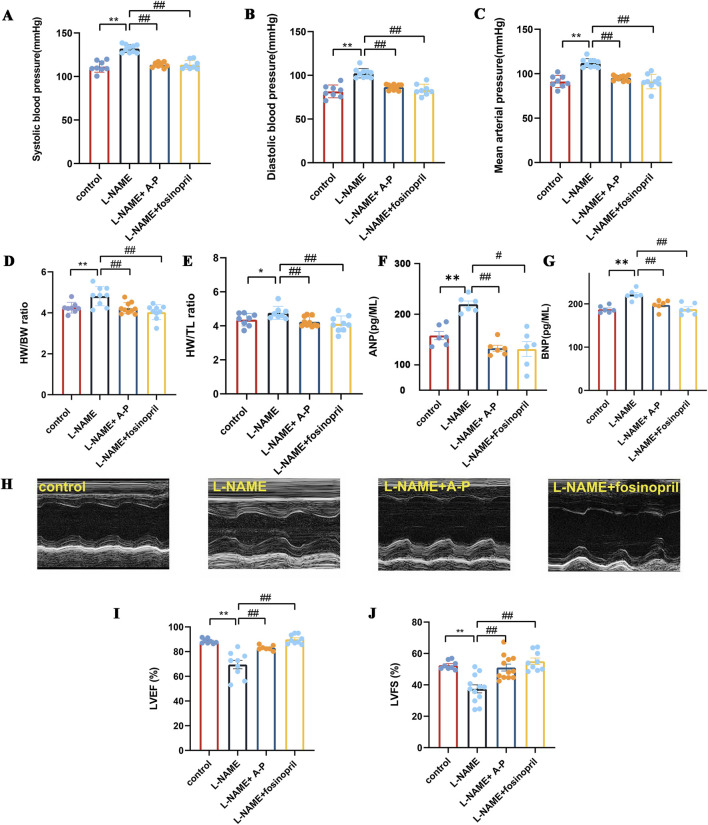
Effects of AB-PL on blood pressure, cardiac hypertrophy, and cardiac functions in L-NAME-induced hypertensive mice; **(A)** systolic blood pressure (SBP); **(B)** diastolic blood pressure (DBP); **(C)** mean arterial pressure (MAP); **(D)** heart weight to bodyweight (HW/BW) ratio; **(E)** heart weight to tibia length (HW/TL) ratio; **(F)** atrial natriuretic peptide (ANP) and **(G)** brain natriuretic peptide (BNP) expression levels; **(H)** representative echocardiographic images; **(I)** left ventricular ejection fraction (LVEF); **(J)** left ventricular fractional shortening (LVFS) (n ≥ 3; ^#^ *p* < 0.05, ^##^ *p* < 0.01 vs. model group; ^*^ *p* < 0.05, ^**^ *p* < 0.01 vs. control group).

To assess the effects of L-NAME on blood pressure injury, we first assessed the blood pressure values of the mice. Mice receiving a gavage of L-NAME showed significant increases in the systolic blood pressure, diastolic blood pressure, and mean arterial pressure compared to the control group, and these increases were inhibited upon treatment with AB-PL (Figures 2A–C). Cardiac hypertrophy was determined by measuring the HW/BW and HW/TL ratios in the mice. It was apparent that the HW/BW ratios of the L-NAME group were significantly higher than those of the control group (*p* < 0.01). Additionally, the HW/BW and HW/TL ratios in mice receiving AB-PL and fosinopril treatments were significantly lower than those of the control group (*p* < 0.01) (Figures 2D,E). The atrial natriuretic peptide (ANP) and brain natriuretic peptide (BNP) levels in the L-NAME group were significantly higher than those in the control group (*p* < 0.05); in addition, mice treated with AB-PL had significantly lower ANP and BNP levels than the control group (Figures 2F,G). The left ventricular end-diastolic diameter (LVEDd) and left ventricular end-systolic diameter (LVEDs) of the L-NAME group were larger than those of the control and AB-PL groups based on visual observations of the echocardiography images (Figure 2H). The left ventricular ejection fraction (LVEF) and left ventricular fractional shortening (LVFS) of the L-NAME group were significantly decreased (*p* < 0.01), and AB-PL improved both these parameters compared to the L-NAME group (*p* < 0.05) (Figures 2I,J).

### 3.3 Effects of AB-PL treatment on components related to nitrogen metabolism

**FIGURE 3:**
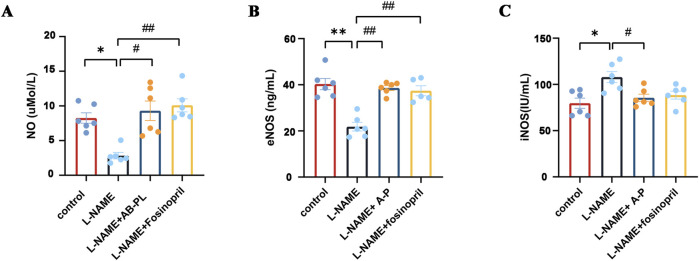
Effects of AB-PL on key components of the nitrogen metabolism pathway in L-NAME-induced hypertensive mice: **(A)** nitric oxide (NO), **(B)** endothelial nitric oxide synthase (eNOS), and **(C)** inducible nitric oxide synthase (iNOS) levels (n ≥ 3; ^#^ *p* < 0.05, ^##^ *p* < 0.01 vs. model group; ^*^ *p* < 0.05, ^**^ *p* < 0.01 vs. control group).

We investigated the effects of AB-PL on the expression of NO, eNOS, and iNOS in the nitrogen metabolic pathways in L-NAME-induced mice. The serum levels of NO, eNOS, and iNOS following 8 weeks of intragastric administration of L-NAME are shown in Figures 3A–C; these levels were significantly increased in all experimental groups compared to the model group (*p* < 0.05, *p* < 0.01). The results show that AB-PL treatment increases the levels of NO and eNOS while decreasing the level of iNOS.

### 3.4 Effects of AB-PL treatment on oxidative stress and the Nrf2/HO-1 pathway

**FIGURE 4:**
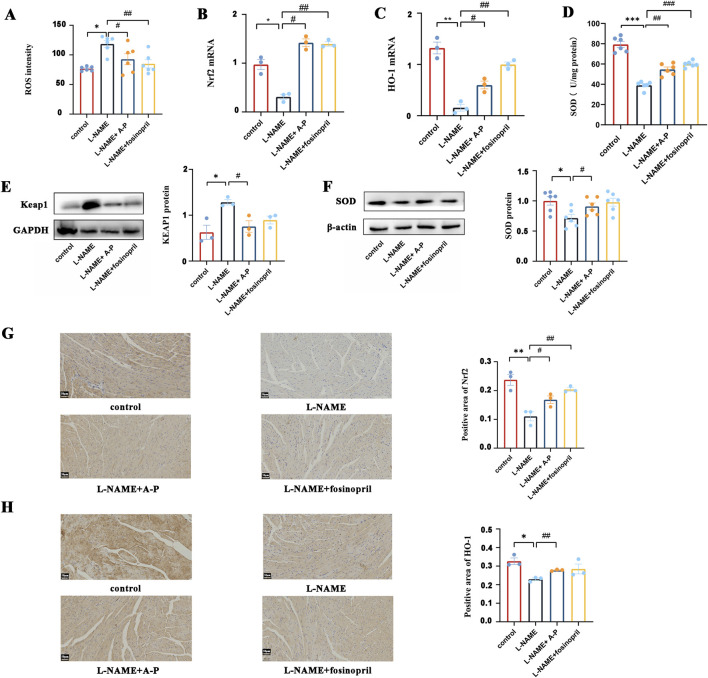
Effects of AB-PL on oxidative stress and the Nrf2/HO-1 signaling pathway in the myocardial tissues of hypertensive mice: **(A)** reactive oxygen species (ROS) level; **(B)** Nrf2 and **(C)** HO-1 mRNA expression; **(D)** superoxide dismutase (SOD) enzyme activity; **(E)** Keap1, and **(F)** SOD protein expression. Immunohistochemical staining of **(G)** Nrf2 and **(H)** HO-1 (n ≥ 3; ^#^ *p* < 0.05, ^##^ *p* < 0.01 vs. model group; ^*^ *p* < 0.05, ^**^ *p* < 0.01 vs. control group).

We evaluated the effects of AB-PL on oxidative stress, specifically its impacts on the Nrf2/HO-1 signaling pathway in L-NAME-induced hypertensive mice; fluorescent probe detection showed elevated ROS levels in the myocardial tissues of the L-NAME group relative to the controls, whereas AB-PL treatment markedly reduced ROS levels (Figure 4A). Using RT-qPCR and Western blotting, we analyzed the mRNA and protein levels of the key pathway components in the myocardium after 8 weeks of L-NAME administration. Compared to the controls, the L-NAME group exhibited significantly reduced mRNA expression of Nrf2 and HO-1, along with decreased SOD protein expression and increased Keap1 protein levels. Furthermore, AB-PL treatment effectively alleviated these changes by upregulating the expression of Nrf2 and HO-1 as well as modulating the Keap1 and SOD levels (Figures 4B,C,E,F). Using RT-qPCR and Western blotting, we analyzed the mRNA and protein levels of key pathway components in the myocardium after 8 weeks of L-NAME administration. Immunohistochemical staining revealed cytoplasmic localization of Nrf2 and HO-1 in cardiomyocytes, while semiquantitative analysis indicated that the L-NAME group had significantly lower levels of Nrf2 and HO-1 immunostaining positivity compared to the AB-PL-treated group (Figures 4G,H). Collectively, these results demonstrate that AB-PL alleviates oxidative stress by activating the Nrf2/HO-1 pathway and reducing ROS level in the hypertensive myocardium.

### 3.5 AB-PL attenuates inflammation marker expression in L-NAME murine myocardium

**FIGURE 5:**
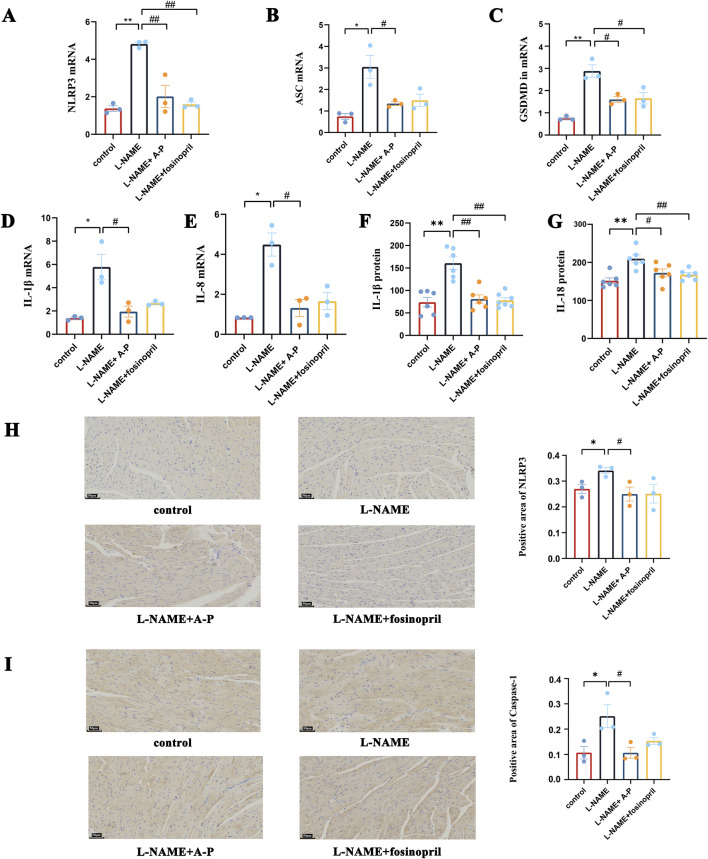
Effects of AB-PL on the NLRP3 inflammasome pathway and downstream inflammatory markers in the myocardial tissues of hypertensive mice: mRNA expression levels of **(A)** NLRP3, **(B)** ASC, **(C)** gasdermin D (GSDMD), **(D)** IL-1β, and **(E)** IL-18; protein levels of **(F)** IL-1β and **(G)** IL-18; immunohistochemical staining of **(H)** NLRP3 and **(I)** caspase-1 proteins (n = 3; ^#^ *p* < 0.05, ^##^ *p* < 0.01 vs. model group; ^*^ *p* < 0.05, ^**^ *p* < 0.01 vs. control group).

To validate the effects of AB-PL on the inflammatory cytokines induced by L-NAME, the NLRP3 inflammasome and downstream inflammatory factors were detected after L-NAME gavage for 8 weeks. We detected markers of the NLRP3 inflammasome pathway, including NLRP3, ASC, gasdermin D (GSDMD), IL-1β, and IL-18, in the murine myocardial tissues by RT-qPCR; these inflammatory markers were increased in the L-NAME group, indicating activation of myocardial inflammation induced by pressure overload. Thus, AB-PL mitigates increases in the levels of NLRP3, ASC, GSDMD, IL-1β, and IL-18 (Figures 5A–E). The ELISA evaluations for IL-1β and IL-18 protein levels showed similar results as RT-qPCR (Figures 5F,G), namely, that AB-PL attenuates inflammatory responses in L-NAME mice. Immunohistochemical staining revealed that NLRP3 was localized in the cytoplasm of cardiomyocytes; the results show increased levels of NLRP3 and caspase-1 in the L-NAME group than the AB-PL group (Figures 5H,I).

### 3.6 Effects of AB-PL treatment on lipid metabolism in mice

**FIGURE 6:**
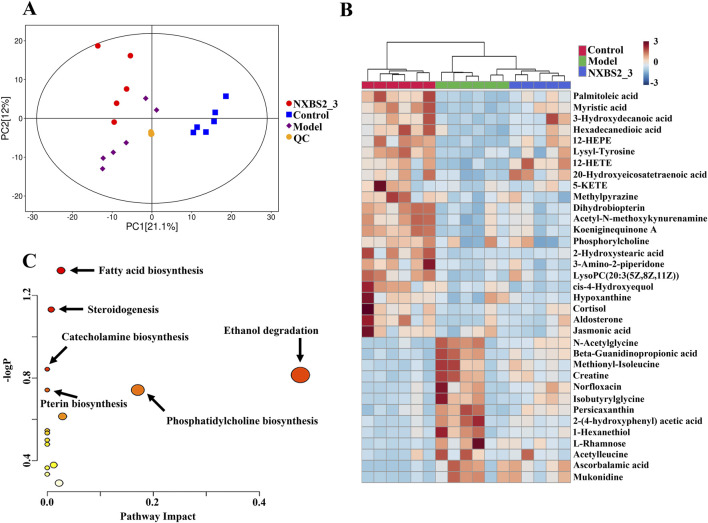
Metabolic profiling and pathway analyses of serum samples from different treatment groups: **(A)** principal component analysis (PCA) score plot including quality control (QC) samples; **(B)** heatmap of representative metabolites among the control, model, and AB-PL groups; **(C)** key altered metabolic pathways.

As shown in Figure 6A, the quality control (QC) samples were tightly clustered in the principal component analysis, demonstrating the reliability of the metabolomic method. Further, there were remarkable disparities in the metabolic profiles of the samples across different treatment groups, indicating variations in the protective effects of different drug combinations. Finally, the key metabolites used for intergroup categorization were screened according to the following criteria: variable importance projection >1, *p* < 0.05, and |log_2_(fold change)| > 0. A total of 35 metabolites were found to be significantly altered in the serum samples from the L-NAME group compared to the control group, while AB-PL treatment significantly restored the expression of these common metabolites to normal levels to varying degrees (Figure 6B; Table 1). Metabolic pathway analysis was then performed by importing these differential metabolites into MetaboAnalyst (http://www.metaboanalyst.ca/) to explore the possible mechanisms through which AB-PL regulates cardiac structure and functions. By setting the screening threshold as raw *p* < 0.05, we identified six key metabolic pathways, including fatty acid biosynthesis, steroidogenesis, catecholamine biosynthesis, ethanol degradation, pterin biosynthesis, and phosphatidylcholine biosynthesis (Figure 6C).

**TABLE 1:** Potential differentiated biomarkers screened from the serum samples of mice.

Biomarker	MS2 score	Level	mzmed
(±)-Jasmonic acid	0.649171077	B(i)	209.1176099
12-HEPE	0.531518308	B(iii)	317.2121237
12-HETE	0.568255077	B(iii)	319.227761
1-Hexanethiol	0.452069692	B(iii)	119.089528
20-Hydroxyeicosatetraenoic acid	0.792508077	B(i)	319.227545
2-Hydroxystearic acid	0.980654538	B(ii)	299.2590744
3-Amino-2-piperidone	1	B(ii)	115.0866173
3-Hydroxydecanoic acid	0.623899846	B(i)	187.1332825
5-KETE	0.868534385	B(ii)	319.2262464
Acetyl leucine	0.932055615	B(i)	172.097175
Acetyl-N-formyl-5-methoxykynurenamine	0.713927538	B(iii)	259.0682182
Aldosterone	1	B(i)	359.1864902
Ascorbalamic acid	0.680488154	B(iii)	264.0672927
Beta-guanidinopropionic acid	0.992872846	B(ii)	132.0767492
cis-4-Hydroxyequol	0.613149692	B(iii)	259.0918838
Cortisol	0.85103	B(ii)	363.2157241
Creatine	1	B(i)	130.0612583
Dihydrobiopterin	0.498804077	B(iii)	240.1101074
Hexadecanedioic acid	0.519173923	B(iii)	285.206872
Hypoxanthine	0.716105462	B(iii)	137.0455434
Isobutyryl glycine	0.980315692	B(i)	144.0656385
Koeniginequinone A	0.513404769	B(iii)	242.078458
L-Rhamnose	0.899279308	B(i)	209.0660422
LysoPC (20:3 (5Z,8Z,11Z))	0.541552538	B(iii)	546.3538185
Lysyl-tyrosine	0.562220154	B(iii)	310.1754829
Methionyl isoleucine	0.947417538	B(ii)	263.1455669
Methylpyrazine	1	B(ii)	95.06073546
Mukonidine	0.940187385	B(ii)	242.0854187
Myristic acid	0.838683308	B(i)	227.2012655
N-Acetyl-glycine	0.979318923	B(i)	116.0343405
N-Carbamoyl-2-amino-2-(4-hydroxyphenyl) acetic acid	0.575770923	B(iii)	211.0685706
Norfloxacin	0.503313462	B(iii)	320.1346685
Palmitoleic acid	0.650185077	B(iii)	253.2172616
Persicaxanthin	0.472749615	B(iii)	385.2727056
Phosphorylcholine	0.979216308	B(ii)	184.0731745

## 4 Discussion

In this study, our data provide evidence supporting the idea that the AB-PL herb pair exhibits significant therapeutic potential in the treatment of hypertensive cardiomyopathy. The potential action mechanisms were investigated through an integrated framework combining network pharmacology predictions and experimental exploration. The findings suggest that AB-PL exerts its protective effects on hypertensive cardiomyopathy via several putatively interconnected biological pathways, including nitric oxide metabolism, oxidative stress, inflammation, and lipid metabolism.

To establish a comprehensive correlation between the network pharmacology predictions and our experimental observations, we first identified the key bioactive compounds in AB-PL (Supplementary Table S2). Then, we linked these compounds with the mechanistically implicated pathways. For instance, quercetin and berberine as the two major bioactive compounds present in *A. bidentata* Blume were predicted to target pathways related to oxidative stress (Nrf2/HO-1) and inflammation (NLRP3 inflammasome) (Li et al., 2024; Yiyang et al., 2023; Linfeng et al., 2022; Hongxia et al., 2021). Further, our experimental observations showed that AB-PL treatment significantly upregulated Nrf2 and HO-1 protein expression (Figure 4) while inhibiting NLRP3 inflammasome activation along with subsequent secretion of IL-1β and IL-18 (Figure 5). Next, paeoniflorin as a characteristic monoterpenoid glycoside from *P. lactiflora* Pall. has been previously reported to exert effects against cardiac hypertrophy, which aligns with our current findings that AB-PL ameliorates cardiac remodeling and dysfunction in hypertensive cardiomyopathy (Ren et al., 2023). The coordinated effects of these herb-based compounds administered orally as AB-PL granules underlie the observed multitarget therapeutic effects, bridging the *in silico* predictions with the *in vivo* outcomes.

Nitric oxide metabolism might play an important regulatory role in hypertensive states and mainly involves changes in nitric oxide (NO), endothelial nitric oxide synthase (eNOS), and inducible nitric oxide synthase (iNOS) expression levels (Tran et al., 2022; Pérez-Torres et al., 2020). Nitric oxide is a key vasodilatory molecule capable of lowering blood pressure by activating guanylate cyclase, leading to smooth muscle cell relaxation and vasodilation (Ataabadi et al., 2020). Our data suggest that AB-PL could attenuate L-NAME-induced NO reduction through modulation of the nitric oxide metabolism pathways (Figure 3A). eNOS is the main enzyme responsible for the synthesis of NO in endothelial cells, and a decrease in its activity might affect vasodilatory function to further exacerbate hypertension. Meanwhile, increased expression of inducible iNOS in hypertensive states lead to a transient increase in the NO level, which promotes the onset and development of inflammation to cause abnormalities in the vascular and cardiovascular systems.

Nrf2 has been widely recognized as the key transcription factor responsible for controlling the activities of key antioxidant factors, including HO-1 (Loboda et al., 2016). Nrf2 pathway activation occurs under stressful conditions and is believed to be essential for sensing oxidative stress and protecting cells against ROS (Pogu et al., 2019; Jia et al., 2022). Nrf2 had been reported to exert potential protective roles in myocardial ischemia and reperfusion injury (Xu et al., 2019), acute kidney injury (Qiu et al., 2022), and vascular endothelial damage (Yang et al., 2019). Furthermore, Nrf2 had been shown to attenuate inflammation in smoking-induced chronic obstructive pulmonary disease/emphysema and asthma (Sakurai et al., 2018; Cui et al., 2018). Our findings also revealed that the NLRP3 inflammasome, which plays a pivotal role in the inflammatory cascade, was significantly inhibited following AB-PL treatment; this inhibition led to reduced levels of proinflammatory cytokines like IL-1β and IL-18, suggesting that AB-PL may exert antihypertensive effects by modulating the inflammatory pathways contributing to vascular dysfunction.

Studies have suggested that the development of hypertensive cardiomyopathy is associated with inflammatory factors, of which the NLRP3 inflammasome is closely implicated in the pathogenesis of hypertensive cardiomyopathy. Activation of the NLRP3 inflammasome triggers caspase-1 activation and subsequent secretion of IL-1β and IL-18, which exacerbate the inflammatory responses (Hu et al., 2024). Dalekos et al. (1997) found that the serum levels of IL-1β were significantly elevated in patients with essential hypertension, indicating a correlation between the inflammatory mediator IL-1β and hypertension. Pharmacological inhibition of NLRP3 inflammasomes has been reported to reduce blood pressure, renal injury, and renal dysfunction in salt-sensitive hypertension (Krishnan et al., 2019). It was also reported that NLRP3 inflammasomes contribute to the development of gestational hypertension (Shirasuna et al., 2016). Further, there is evidence that NLRP3 inflammasomes are expressed in the endothelial cells and that their activation causes endothelial dysfunction (Bai et al., 2020). Our findings indicate that L-NAME administration activates the NLRP3 inflammasomes; in contrast, administration of AB-PL inhibits NLRP3 inflammasomes, suppresses their associated inflammatory signaling pathways, and attenuates the inflammatory responses by the body.

Metabolomic analysis revealed associations between AB-PL treatment and alterations in the lipid metabolism pathways, such as fatty acid biosynthesis and steroidogenesis. These observations are consistent with the network pharmacology predictions that initially identified lipid metabolism as a potential pathway of interest. It is important to note that these metabolomic changes represent correlative signatures observed alongside the therapeutic efficacy of AB-PL. Although the regulation of lipid metabolism is critically involved in hypertension, affecting endothelial functions and vascular tone, the present findings do not establish that modulation of these specific metabolic pathways is a direct causal mechanism of AB-PL’s actions. Instead, our findings provide valuable hypotheses for future research to determine whether these metabolic shifts drive the therapeutic effects or are secondary consequences of the improved physiological state.

In addition to the physiological regulation of water, sodium, and potassium homeostasis, aldosterone is known to regulate several physiological and pathological processes in the cardiovascular system. At the vascular level, excess aldosterone stimulates endothelial dysfunction and inflammatory cell infiltration, enhances the development of atherosclerotic plaques, and favors plaque instability, arterial stiffness, and calcification. At the cardiac level, aldosterone increases cardiac inflammation, fibrosis, and cardiac hypertrophy (Buffolo et al., 2022). The biomarker 20-hydroxy-5,8,11,14-eicosatetraenoic acid (20-HETE) has been reported to play important roles in vasoconstriction, autoregulation, endothelial dysfunction, angiogenesis, inflammation, and blood–brain barrier integrity and has also been implicated in hypertension; 20-HETE enhances vascular responses to vasoconstrictors to promote hypertension and lowers blood pressure by inhibiting sodium transport in the kidney (Shekhar et al., 2019). Studies have indicated that nutmeg stimulates cellular apolipoprotein III expression and increases apolipoprotein III (ApoCIII) concentration, which may contribute to atherosclerosis, coronary heart disease, hypertriglyceridemia, and type II diabetes (Olivieri et al., 2020; Rocha et al., 2017). Circulating palmitoleic acid (POA) is inversely associated with risk of primary hypertension, and exogenous POA supplementation has been shown to decrease the systolic blood pressure while improving aortic remodeling by inhibiting NF-κB-mediated inflammation (Tang et al., 2021). Phosphorylcholine (PC) is an epitope on oxidized low-density lipoprotein (LDL), dead cells, and some microorganisms; it is positively associated with various chronic inflammatory diseases, including atherosclerosis, cardiovascular disease, rheumatic disease, and chronic kidney disease. Oxidized LDL plays a proinflammatory role in the development of atherosclerosis, and PC exposed on the surface of LDL during oxidation may play an important role in oxidized-LDL-induced immune activation (Jujić et al., 2021). At the same time, studies have shown that phosphocholine generates oxidized phosphatidylcholine, such as 1-palmitoyl-2-(5-oxovaleroyl)-sn-glycero-3-phosphocholine, to induce activation of NLRP3 inflammasomes and result in IL-1β production in macrophages (Yeon et al., 2017).

Our findings suggest intricate interrelationships among the nitrogen metabolic pathway, Nrf2/HO-1 signaling pathway, NLRP3 inflammatory pathway, and lipid metabolism to potentially form a cellular regulatory network. Nitric oxide (NO) in the nitrogen metabolic pathway is known to have an important signaling function in cells, and NO production is closely related to eNOS activity. Studies have shown that IL-1β downregulates eNOS phosphorylation by inhibiting Akt expression (Xing et al., 2019). IL-1β also impairs eNOS expression and activity by enhancing oxidative stress. Additionally, knockdown of NLRP3 significantly attenuates AngII-induced vascular oxidative stress. Several studies have shown that the Nrf2/HO-1 signaling pathway plays a key role in the regulation of oxidative stress and that Nrf2 inhibits activation of the NLRP3 inflammatory vesicles by controlling the Trx1/TXNIP complex (Hou et al., 2018). Chen et al. (2019) reported that inhibition of Nrf2/HO-1 signaling in osteoarthritis enhanced NLRP3 inflammatory vesicle signaling. Moreover, reactive oxidants like ROS are believed to be key regulators of NLRP3 activation. Increased ROS might contribute to the activation of NLRP3, leading to the initiation of inflammatory responses (Qiu et al., 2019; Minutoli et al., 2016). Together, the Nrf2/HO-1 signaling pathway may regulate the NLRP3 inflammatory pathway by inhibiting oxidative stress. Further, nitric oxide from the nitrogen metabolism pathway and oxidized phosphatidylcholine from lipid metabolism may also be involved in NLRP3 activation; these potential interactions might help maintain nitrogen metabolism and redox homeostasis within the cells, playing important mediating roles in the regulation of inflammation.

Given the key findings of this study, we acknowledge several limitations that should be addressed in future research. First, direct functional validation of the predicted core pathways was not performed. Although we identified nitrogen metabolism, Nrf2/HO-1 signaling, NLRP3 inflammasome, and lipid metabolism as the targets of AB-PL treatment via multiomics analyses, we lack specific evaluations with Nrf2/NLRP3 knockout mice or pharmacological inhibitors (e.g., MCC950 for NLRP3) to confirm that these pathways are required for the effects of AB-PL. Our conclusions reflect the correlations among AB-PL, molecular changes, and phenotype improvements rather than definite cause–effect relationships. Second, the animal sample size used herein was modest; although this was sufficient to detect major changes (e.g., blood pressure, LVEF), it could weaken the statistical power for subtle shifts (e.g., some low-abundance metabolites). We mitigated this constraint with three independent biological replicates but larger cohorts will further strengthen the robustness of the secondary molecular endpoints.

We integrated three complementary approaches, namely, network pharmacology, *in vivo* experimentation, and metabolomics analysis, in this study to avoid biases inherent to any single method. Network pharmacology initially allowed prediction of the core pathways involved in the actions of AB-PL. In subsequent *in vivo* tests, we verified that AB-PL restored serum NO levels, upregulated Nrf2 expression, and suppressed NLRP3 activation. Finally, metabolomic analysis enabled extension of these findings through the identification of 35 differentially abundant metabolites and implication of lipid-related pathways, thereby integrating the predictive and experimental layers of evidence into a cohesive investigative framework. Next, AB-PL was found to improve the relevant phenotypes; it lowered systolic blood pressure, reversed cardiac hypertrophy, and boosted LVEF, along with normalization of ANP/BNP and IL-1β/IL-18 levels, supporting that idea that the molecular effects of AB-PL translate to real physiological benefits.

**FIGURE 7:**
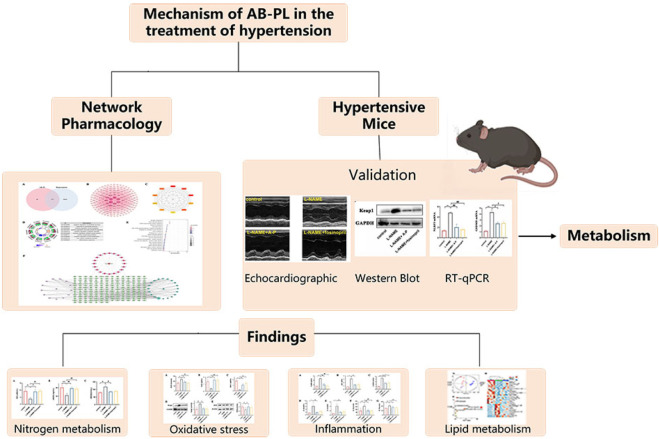
Flowchart of the mechanistic investigation of AB-PL in the treatment of hypertensive cardiomyopathy.

In conclusion, the results of this study provide comprehensive insights into the multifaceted potential mechanisms through which AB-PL may exert its antihypertensive effects, primarily by modulating nitric oxide metabolism, oxidative stress, and inflammation. The metabolomic findings offer supplementary insights and novel hypotheses regarding associated metabolic alterations. The findings position AB-PL as a promising therapeutic candidate for the treatment of hypertension, demonstrating a novel methodological approach integrating network pharmacology predictions with experimental investigations (Figure 7). This work not only contributes to improved understanding of the pharmacological basis of AB-PL therapy but also lays the preliminary groundwork for potential clinical applications of traditional Chinese medicine in the management of hypertension.

## Data Availability

The original contributions presented in the study are included in the article/Supplementary Material, and any further inquiries may be directed to the corresponding authors.
