# Supplementary material for: Multitarget mechanisms of the herb pair Achyranthes bidentata and Paeonia lactiflora Pall. in ameliorating hypertensive cardiomyopathy: combining network pharmacology and functional exploration
Source: Front Pharmacol. 2026 Feb 11;17:1717533. doi: 10.3389/fphar.2026.1717533 (PMC12932230; doi:10.3389/fphar.2026.1717533)
Supplement: Supplementary file 1 [file Supplementaryfile1.docx]

**
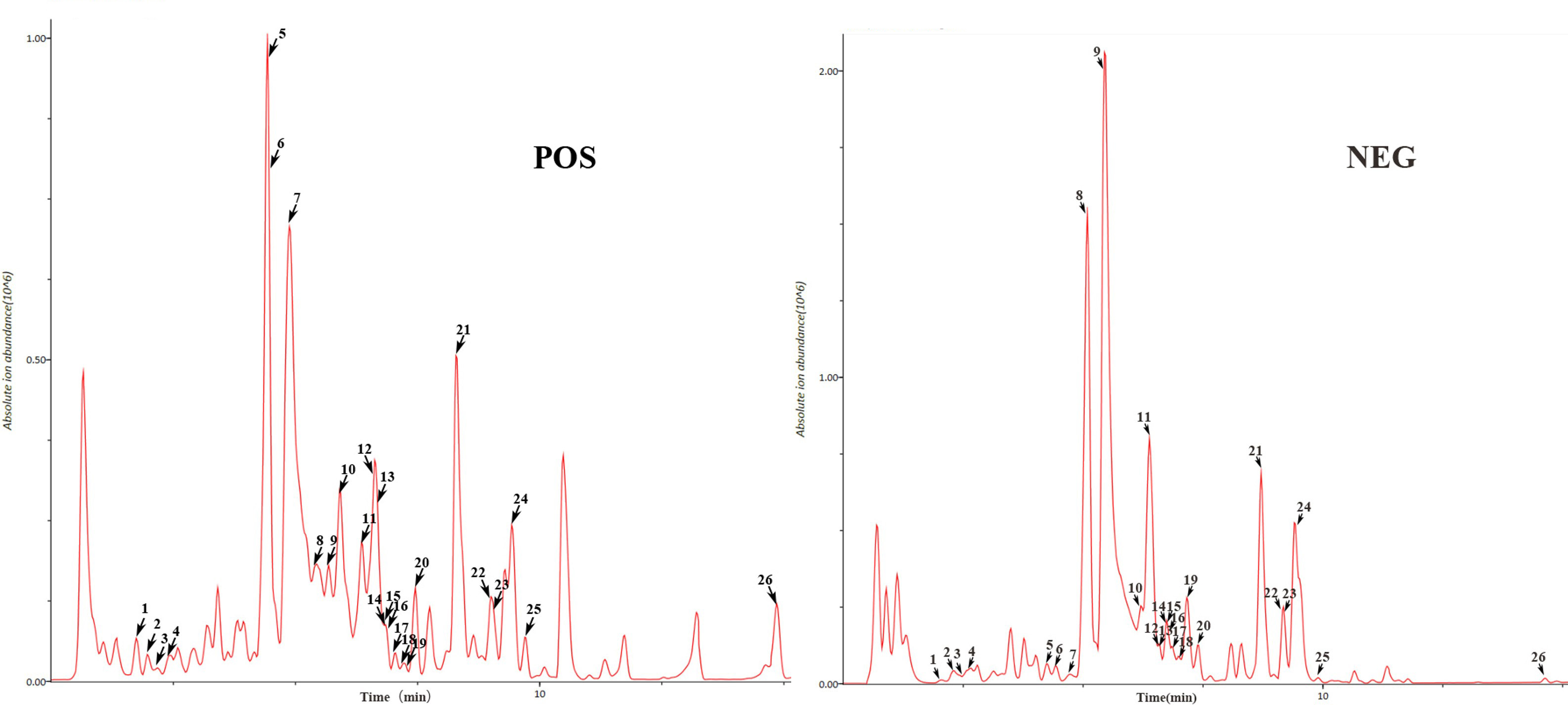
**

**Fig.S1** The total ion chromatogram of AB-PL in positive (A) and negative (B) mode.

**Table. S1** Information of AB-PL major components. Mass Error of components were not greater than 10 ppm.

| NO. | Compound | Formula | t_R_/ min |
| --- | --- | --- | --- |
| 1 | [Chrysophanic acid](https://www.chemsrc.com/en/cas/481-74-3_389949.html) | C_15_H_10_O_4_ | 1.6 |
| 2 | Palmitic acid | C_16_H_32_O_2_ | 2.0 |
| 3 | benzoic acid | C_7_H_6_O_2_ | 2.2 |
| 4 | Mudanpioside C | C_30_H_32_O_13_ | 2.4 |
| 5 | 6'-O-Galloyl paeoniflorin | C_30_H_32_O_15_ | 4.1 |
| 6 | Paeonolide | C_20_H_28_O_12_ | 4.3 |
| 7 | Naringenin | C_15_H_12_O_5_ | 4.9 |
| 8 | Cyasterone | C_29_H_44_O_8_ | 5.2 |
| 9 | Gallic acid | C_7_H_6_O_5_ | 5.3 |
| 10 | Lactiflorin | C_23_H_26_O_10_ | 5.6 |
| 11 | Benzoylpaeoniflorin | C_30_H_32_O_12_ | 5.8 |
| 12 | albiflorin | C_23_H_28_O_11_ | 6.0 |
| 13 | β-ecdysterone | C_27_H_44_O_7_ | 6.0 |
| 14 | Paeoniflorin | C_23_H_28_O_11_ | 6.1 |
| 15 | 25-Inokosterone | C_27_H_44_O_7_ | 6.1 |
| 16 | Oxypaeoniflorin | C_23_H_28_O_12_ | 6.1 |
| 17 | Paeonol | C_9_H_10_O_3_ | 6.5 |
| 18 | alpha-Spinasterol | C_29_H_48_O | 6.7 |
| 19 | 5-hydroxymethylfurfural | C_6_H_6_O_3_ | 6.8 |
| 20 | Benzoylpaeoniflorin | C_30_H_32_O_12_ | 7.6 |
| 21 | Tocopherols | C_29_H_50_O_2_ | 8.5 |
| 22 | Chikusetsusaponin IVa | C_42_H_66_O_14_ | 9.0 |
| 23 | Ginsenoside Ro | C_48_H_76_O_19_ | 9.0 |
| 24 | Nobiletin | C_21_H_22_O_8_ | 9.5 |
| 25 | 1,2,3,4,6-O-Pentagalloylglucose | C_41_H_32_O_26_ | 10.1 |
| 26 | Beta-Sitosterol | C_30_H_52_O | 20.7 |

**Table.S2**  Primer sequences of the target genes

| Gene | primer sequence |
| --- | --- |
| Nrf2-F | 5’-GCCACCGCCAGGACTACAG-3’ |
| Nrf2-R | 5’-GCAACAAGAGCAGCCACCTC-3’ |
| HO-1-F | 5’-AGACCGCCTTCCTGCTCAAC-3’ |
| HO-1-R | 5’-GACGAAGTGACGCCATCTGTG-3’ |
| NLRP3-F | 5’-GCTGCGATCAACAGGCGAGAC-3’ |
| NLRP3-R | 5’-CCATCCACTCTTCTTCAAGGCTGTC-3’ |
| ASC-F | 5’-GGACGGAGTGCTGGATGCTTTG-3’ |
| ASC-R | 5’-CATCTTGTCTTGGCTGGTGGTCTC-3’ |
| GSDMD-F | 5’-ACTGAGGTCCACAGCCAAGAGG-3’ |
| GSDMD-R | 5’-GCCACTCGGAATGCCAGGATG-3’ |
| IL-1β-F | 5’-CACTACAGGCTCCGAGATGAACAAC-3’ |
| IL-1β-R | 5’-TGTCGTTGCTTGGTTCTCCTTGTAC-3’ |
| IL-18-F | 5’-CAAAGTGCCAGTGAACCCCAGAC-3’ |
| IL-18-R | 5’-ACAGAGAGGGTCACAGCCAGTC-3’ |
| GAPDH-F | 5’-CACCATCTTCCAGGAGCGAGAC-3’ |
| GAPDH-R | 5’-GACACCAGTAGACTCCACGACATAC-3’ |

**Table.S3** AB-PL active ingredient. **^#^**, components have been verified and identified by LC-MS.

| medicinal herb | MOL ID | Main active ingredients | OB% | DL |
| --- | --- | --- | --- | --- |
| Achyranthes bidentata Blume | MOL000785 | palmatine | 64.6 | 0.65 |
| Achyranthes bidentata Blume | MOL000098 | quercetin | 46.43 | 0.28 |
| Achyranthes bidentata Blume | MOL012542 | β-ecdysterone**^#^** | 44.23 | 0.82 |
| Achyranthes bidentata Blume | MOL000449 | Stigmasterol | 43.83 | 0.76 |
| Achyranthes bidentata Blume | MOL002897 | epiberberine | 43.09 | 0.78 |
| Achyranthes bidentata Blume | MOL001006 | poriferasta-7,22E-dien-3beta-ol | 42.98 | 0.76 |
| Achyranthes bidentata Blume | MOL004355 | Spinasterol**^#^** | 42.98 | 0.76 |
| Achyranthes bidentata Blume | MOL000422 | kaempferol | 41.88 | 0.24 |
| Achyranthes bidentata Blume | MOL012537 | Spinoside A | 41.75 | 0.4 |
| Achyranthes bidentata Blume | MOL002776 | Baicalin | 40.12 | 0.75 |
| Achyranthes bidentata Blume | MOL003847 | Inophyllum E | 38.81 | 0.85 |
| Achyranthes bidentata Blume | MOL002643 | delta 7-stigmastenol | 37.42 | 0.75 |
| Achyranthes bidentata Blume | MOL000358 | beta-sitosterol | 36.91 | 0.75 |
| Achyranthes bidentata Blume | MOL000085 | beta-daucosterol_qt | 36.91 | 0.75 |
| Achyranthes bidentata Blume | MOL001454 | berberine | 36.86 | 0.78 |
| Achyranthes bidentata Blume | MOL012461 | 28-norolean-17-en-3-ol | 35.93 | 0.78 |
| Achyranthes bidentata Blume | MOL002714 | baicalein | 33.52 | 0.21 |
| Achyranthes bidentata Blume | MOL012505 | bidentatoside | 31.76 | 0.59 |
| Achyranthes bidentata Blume | MOL000173 | wogonin | 30.68 | 0.23 |
| Achyranthes bidentata Blume | MOL001458 | coptisine | 30.67 | 0.86 |
| Paeonia lactiflora Pall | MOL001918 | paeoniflorgenone | 87.59 | 0.37 |
| Paeonia lactiflora Pall | MOL001925 | paeoniflorin_qt | 68.18 | 0.4 |
| Paeonia lactiflora Pall | MOL001928 | albiflorin**^#^** | 66.64 | 0.33 |
| Paeonia lactiflora Pall | MOL001910 | 11alpha,12alpha-epoxy-3beta-23-dihydroxy-30-norolean-20-en-28,12beta-olide | 64.77 | 0.38 |
| Paeonia lactiflora Pall | MOL000211 | Mairin | 55.38 | 0.78 |
| Paeonia lactiflora Pall | MOL000492 | (+)-catechin | 54.83 | 0.24 |
| Paeonia lactiflora Pall | MOL001924 | paeoniflorin**^#^** | 53.87 | 0.79 |
| Paeonia lactiflora Pall | MOL001921 | Lactiflorin**^#^** | 49.12 | 0.8 |
| Paeonia lactiflora Pall | MOL001919 | (3S,5R,8R,9R,10S,14S)-3,17-dihydroxy-4,4,8,10,14-pentamethyl-2,3,5,6,7,9-hexahydro-1H-cyclopenta[a]phenanthrene-15,16-dione | 43.56 | 0.53 |
| Paeonia lactiflora Pall | MOL000422 | kaempferol | 41.88 | 0.24 |
| Paeonia lactiflora Pall | MOL000358 | beta-sitosterol**^#^** | 36.91 | 0.75 |
| Paeonia lactiflora Pall | MOL000359 | Sitosterol | 36.91 | 0.75 |
| Paeonia lactiflora Pall | MOL001930 | benzoyl paeoniflorin**^#^** | 31.27 | 0.75 |

**Table. S4 The target information corresponding to AB-PL and hypertensive cardiomyopathy**

| Name | Gene ID | UniProt ID | Name | Gene ID | UniProt ID |
| --- | --- | --- | --- | --- | --- |
| AR | 367 | P10275 | F2 | 2147 | P00734 |
| CYP19A1 | 1588 | P11511 | SIGMAR1 | 10257 | Q99720 |
| SHBG | 6462 | P04278 | SERPINA6 | 5267 | P08185 |
| GABBR1 | 2550 | P34903 | TNF | 7124 | P01375 |
| NR3C1 | 2908 | P08235 | NOS2 | 4843 | P35225 |
| ESR1 | 2099 | P03372 | NR3C2 | 4307 | Q03135 |
| CHRM1 | 1132 | P11229 | PRKCH | 5583 | P17251 |
| LGALS3 | 3956 | P17931 | BACE1 | 2362 | P56817 |
| LGALS9 | 3961 | P47922 | SRD5A2 | 6716 | P28535 |
| PTAFR | 5722 | P25025 | HTR2B | 3356 | P41595 |
| HSPA8 | 3312 | P11142 | CYP2D6 | 1558 | P10635 |
| HSPA5 | 3309 | P11021 | RAC1 | 5879 | P63000 |
| ADORA3 | 135 | P29274 | CDC42 | 1098 | P60953 |
| ADK | 132 | P51573 | RPS6KB1 | 6198 | P42345 |
| PPM1A | 5494 | Q16543 | ADRA2C | 148 | P08913 |
| MME | 4311 | P08473 | AURKA | 6790 | P34066 |
| ECE1 | 1889 | P42858 | AURKB | 9212 | Q99754 |
| ADORA2A | 136 | P29275 | ADRA2B | 147 | P08912 |
| EGFR | 1956 | P00533 | CYP11B2 | 1585 | P15538 |
| SLC6A3 | 6531 | P23975 | CHRM4 | 1137 | P08173 |
| SLC29A1 | 6572 | Q9Y2R7 | TBXAS1 | 6916 | P24557 |
| F3 | 2152 | P08572 | XBP1 | 7494 | Q9Y238 |
| HSP90AA1 | 3320 | P07900 | IKBKB | 3551 | Q9Y6K9 |
| SLC6A2 | 6530 | P23974 | PTGS2 | 5743 | P35354 |
| SSTR5 | 6754 | P30872 | OPRD1 | 4988 | P41145 |
| SSTR2 | 6753 | P30874 | KIT | 3815 | P10721 |
| SSTR4 | 6755 | P30873 | NTRK2 | 4915 | P21588 |
| SSTR1 | 6752 | P30875 | ALOX15 | 241 | P16050 |
| SSTR3 | 6756 | P30876 | CDK1 | 983 | P06493 |
| ABCB1 | 5243 | P08183 | ALOX12 | 242 | P16051 |
| VEGFA | 7422 | P15692 | CA4 | 762 | P13688 |
| FGF1 | 2246 | P05230 | ADORA1 | 134 | P25021 |
| FGF2 | 2247 | P09038 | IL2 | 3558 | P60568 |
| HPSE | 10855 | Q9Y256 | RPS6KA3 | 6197 | P42166 |
| F10 | 2155 | P00742 | ADRA2A | 146 | P08911 |
| SELP | 6403 | P16109 | ALDH2 | 217 | P05091 |
| ADORA2B | 137 | P43080 | HPGD | 3244 | P18413 |
| PRKCA | 5578 | P17252 | GSK3B | 2932 | P49841 |
| MMP13 | 4322 | P45452 | NR1H2 | 7376 | Q9Y255 |
| MMP1 | 4312 | P03956 | CES2 | 1065 | P23141 |
| MMP7 | 4316 | P09238 | PGR | 5241 | P06401 |
| POLB | 5422 | P28340 | CA1 | 759 | P00915 |
| AKR1B10 | 80203 | Q9Y2T8 | CA9 | 764 | P16080 |
| HSD11B1 | 3290 | P08688 | CTSL | 1514 | P07711 |
| PTPN1 | 5770 | P18031 | PREP | 5538 | P14735 |
| PTGES | 5730 | O15296 | THRB | 7068 | P11226 |
| NR1H4 | 9971 | Q9Y2N7 | MAPK14 | 1432 | Q16539 |
| CDC25C | 995 | P30307 | MAP2K1 | 5604 | P28482 |
| PTPN2 | 5771 | P28562 | STAT3 | 6774 | P40763 |
| GPBAR1 | 136064 | Q8IY55 | BCL2L1 | 598 | P10415 |
| TOP2A | 7153 | P11388 | S1PR1 | 1901 | Q16622 |
| CDC25A | 993 | P30304 | CDC25B | 994 | P30305 |
| PTPRF | 5792 | P16885 | PPP1CC | 5499 | P62136 |
| ACP1 | 54 | P05107 | PPP2CA | 5515 | P67775 |
| NPC1L1 | 10062 | Q9Y2I9 | AVPR2 | 554 | P30518 |
| NR1H3 | 10063 | Q9Y2N6 | MAOA | 4128 | P21397 |
| HMGCR | 3156 | P04035 | IGF1R | 3480 | P08069 |
| CYP17A1 | 1586 | P08684 | PIM1 | 5292 | P11309 |
| SREBF2 | 6721 | Q92937 | DRD4 | 1816 | P21917 |
| CYP51A1 | 1595 | P04184 | GLO1 | 2739 | P18419 |
| ESR2 | 2100 | Q92731 | MPO | 4357 | P05164 |
| CYP2C19 | 1557 | P33261 | PIK3R1 | 5295 | P27986 |
| NOX4 | 50507 | Q9NPH2 | DAPK1 | 1612 | Q16586 |
| AKR1B1 | 1041 | P14550 | PYGL | 5836 | P10613 |
| XDH | 7498 | P47895 | SRC | 6714 | P12931 |
| TYR | 7299 | P14679 | PTK2 | 5747 | P11235 |
| FLT3 | 2322 | P36888 | KDR | 3791 | P35968 |
| CA2 | 760 | P00918 | MMP3 | 4314 | P08254 |
| ALOX5 | 240 | P09917 | CA3 | 761 | P00917 |
| CA7 | 766 | P27139 | PLK1 | 5347 | P53350 |
| ABCC1 | 4363 | P33527 | CA6 | 765 | P23613 |
| AHR | 196 | P35869 | MMP9 | 4318 | P14780 |
| CA12 | 763 | P19581 | MMP2 | 4313 | P08253 |
| ESRRA | 2101 | Q15596 | CA14 | 881 | Q16819 |
| CYP1B1 | 1556 | P35569 | CSNK2A1 | 1457 | P19784 |
| ABCG2 | 9429 | Q9UNQ0 | MET | 4233 | P08581 |
| BCHE | 590 | P06276 | NEK2 | 4751 | P51955 |
| CHRM2 | 1135 | P08172 | CXCR1 | 2827 | P25024 |
| SLC6A4 | 6532 | P31645 | CAMK2B | 817 | Q13554 |
| GLI1 | 2735 | P08151 | ALK | 238 | P08068 |
| JUN | 3725 | P05412 | AKT1 | 207 | P31749 |
| PPIA | 5478 | P62937 | PLA2G1B | 5321 | P22307 |
| REN | 5972 | P00797 | AXL | 558 | P30530 |
| HSD11B2 | 3291 | Q03135 | AKR1C2 | 1042 | P28288 |
| ATP1A1 | 476 | P05023 | AKR1C3 | 1043 | P28289 |
| AKR1A1 | 1040 | P17752 | CA13 | 880 | Q9Y2H1 |
| GPR35 | 2852 | Q9H2S0 |  |  |  |

**Metabolomics**

Metabolomics was conducted by Biotree Profile (Shanghai, China). Briefly, 25 mg sample was placed in an EP tube, followed by the addition of 500 μL of an extract solution (methanol: acetonitrile: water = 2: 2: 1, with an isotopically-labelled internal standard mixture). The mixture was homogenized at 35 Hz for 4 minutes and then sonicated for 5 minutes in an ice-water bath. This homogenization and sonication process was repeated three times. Subsequently, the samples were incubated for 1 hour at -40℃ and centrifuged at 12000 rpm for 15 minutes at 4℃. The resulting supernatant was transferred to a new glass vial for analysis. A quality control (QC) sample was prepared by combining equal aliquots of the supernatants from all the samples.

LC-MS/MS analyses were conducted utilizing a UHPLC system (Vanquish, Thermo Fisher Scientific) with a Waters BEH Amide column (2.1 mm × 50 mm, 1.7μm) coupled to an Orbitrap Exploris 120 mass spectrometer (Orbitrap MS, Thermo). The mobile phase, consisting of 25 mmol/L ammonium acetate and 25 ammonia hydroxide in water (pH = 9.75) (A) and acetonitrile (B), facilitated separation. The auto-sampler was maintained at 4 ℃, with an injection volume of 2μL. The Orbitrap Exploris 120 mass spectrometer, employed for its MS/MS capabilities in information-dependent acquisition (IDA) mode, was controlled by Xcalibur software. The ESI source conditions included a sheath gas flow rate of 50 Arb, Aux gas flow rate of 15 Arb, capillary temperature of 350℃, full MS resolution of 60000, MS/MS resolution of 30000, collision energy set at 20/30/40 in NCE mode, and spray voltage at 3 kV (positive) or -3 kV (negative), respectively. The elute gradient was set as follows: 10% B from 0 to 1 min, 10–95% B from 1.01 to 10 min, 95% B from 10.01 to 13 min, and 95–10% B from 13.01 to 15 min.

The initial data in ProteoWizard were transformed into the mzXML format, and subsequent processing involved an in-house program created in R, utilizing XCMS for peak detection, extraction, alignment, and integration. Metabolite annotation employed an in-house MS2 database (BiotreeDB), with an annotation cutoff set at 0.3 for accuracy.
